# Positive changes after a reablement program for older people in the Netherlands: a mixed-methods feasibility study

**DOI:** 10.1186/s12877-026-07460-4

**Published:** 2026-04-23

**Authors:** Elly Branderhorst-Pruijssers, Silke Metzelthin, Ines Mouchaers, Ton Satink, Maud Graff

**Affiliations:** 1 Radboud University Medical Center, IQ Health Scientific Department, Radboudumc, Nijmegen, the Netherlands; 2 Mijzo, Waalwijk, the Netherlands; 3 Department of Health Services Research, Faculty of Health Medicine and Life Sciences, CAPHRI Care and Public Health Research Institute, Maastricht University, Maastricht, the Netherlands; 4 Living Lab in Ageing and Long-Term Care, Maastricht, the Netherlands; 5 Research Group Neurorehabilitation – Self-Regulation and Participation, HAN University of Applied Sciences, Nijmegen, the Netherlands; 6 European Masters of Science in Occupational Therapy, Amsterdam, Netherlands; 7 Radboud University Medical Center, Department of Rehabilitation, Radboudumc, Nijmegen, the Netherlands; 8 Radboud University Medical Center, Radboudumc Alzheimer Center, Nijmegen, the Netherlands

**Keywords:** Reablement, Community health services, Rehabilitation, Older people, Self-reliance, Self-care, Goal-oriented care, Activities of daily living, Health promotion, Patient-centered care, Interdisciplinary care

## Abstract

**Background:**

In recent years, reablement has gained increasing attention as a response to an aging population. The main goal of reablement is to empower older people to engage in and continue to perform the activities that are important to them. The Longer Active at Home (in Dutch: Langer Actief Thuis [LAT]) program is a Dutch reablement program. The aim of this study is to evaluate the feasibility of the LAT program in the Dutch healthcare context.

**Methods:**

A mixed-method study with a pre-/post-study design was conducted evaluating Bowen’s (Am J Prev Med 36:452–7, 2009) five main aspects for feasibility: acceptability, demand, implementation, practicality, and limited efficacy. Qualitative and quantitative data were analyzed simultaneously and triangulated to underlying theoretical concepts. Reflexive thematic analysis was applied to analyze qualitative data from individual interviews with older people and focus group interviews with professionals. The main quantitative outcome was the Canadian Occupational Performance Measure (COPM) to assess self-reliance. Secondary outcomes included an independence score on a Visual Analog Scale and indicated district nursing time. A multilevel linear regression analysis was used to analyze quantitative data.

**Results:**

Seventy-two older people and 15 professionals participated in the study. Regarding demand and acceptability, professionals indicated that LAT aligns with societal trends promoting independence among older people. Furthermore, the new working approach enabled professionals to transition from a “doing for” mindset to a “doing with” mindset, with interdisciplinary collaboration identified as a key success factor—although also a challenge in practice. In terms of limited efficacy, both older people and professionals reported increased self-reliance and self-confidence in the older people. All effectiveness outcomes showed a clinically significant improvement. After the intervention, older people experienced better performance regarding their goals (COPM-P: Δ = 3.10, 95% CI 2.68–3.52) and greater satisfaction with their performance (COPM-S: Δ = 3.18, 95% CI 2.71–3.64). Additionally, they required, on average, 2 h less district nursing care per week.

**Conclusions:**

According to Bowen’s framework, LAT is a feasible intervention. The program enhances older people’s self-reliance and self-confidence, reduces the need for professional care, and supports healthcare professionals in switching from a “doing for” to a “doing with” approach.

**Supplementary Information:**

The online version contains supplementary material available at 10.1186/s12877-026-07460-4.

## Background

In the face of global population aging, the delivery of effective and sustainable care for older people has become a paramount concern. In Europe, the percentage of people aged 65 years and over is increasing rapidly and is expected to reach about 30% of the population by 2050 [1]. In the Netherlands, this is expected to rise from 34% in 2022 to nearly 49% in 2070 [2]. The aging population impacts the Dutch workforce [3], as the shrinking work force, alongside a growing elderly population, will reduce the availability of healthcare professionals. It is therefore important to implement changes in care organization in the Netherlands to ensure that those who really need care can receive it.

Aging is often associated with a decline in physical and cognitive abilities, increasing dependence on others for daily activities [4], such as washing and dressing, household activities, or social activities. Consequently, the demand for health and social care services is rising [5]. Nevertheless, older people wish to remain independent and live in familiar environments for as long as possible [6, 7]. “Aging in place” is increasingly recognized as beneficial for well-being of older people, and to positive experiences in later life [8, 9]. Accordingly, Dutch national healthcare policy emphasizes supporting older people to function independently in their own environment, with technological support, if needed, as described in the Dutch national healthcare policy document Living, Support and Care for the Elderly (in Dutch: Wonen, Ondersteuning en Zorg voor Ouderen [WOZO]) [10].

To support older people living independently, reablement programs have emerged globally as a promising approach to promote independence and to enhance quality of life among older people [11]. WOZO also suggests reablement as a core strategy for promoting self-reliance and reducing and/or preventing the need for support and care [10]. Reablement maximizes an individual’s functional abilities and autonomy in their own environment through goal-oriented interventions. Internationally, it is described as a person-centered, holistic approach aimed at enhancing an individual’s physical and/or other functioning, increasing or maintaining their independence in meaningful daily activities at their place of residence, and reducing long-term service needs [12]. It is delivered by an interdisciplinary team comprising different professionals such as occupational therapists (OTs), physiotherapists (PTs), (district) nurses, and social workers who collaborate based on a goal-oriented plan to support older people in achieving their personal goals [13]. Unlike traditional rehabilitation, which targets deficit remediation, reablement emphasizes relearning skills and strategies to regain or maintain independence in daily activities [14]. Reablement programs have mainly been implemented in home care in the United States, the United Kingdom, Australia, New Zealand, and Scandinavia, with some countries like Denmark and New Zealand integrating it into national policy [12].

Despite growing popularity, evidence on reablement effectiveness is inconclusive. Some studies report significant improvements in independence in daily activities, physical functioning, and quality of life along with a reduced risk of death or institutionalization [15–17], while others report no significant effects [18, 19]. Buma et al. [18], attribute contradictory findings to varying conceptualizations and terminology of reablement; prior to 2015, terms like rehabilitation and restorative care were used interchangeably [14]. Differences in intervention delivery and research methodology further limit generalizability [19]. Consequently, conclusions on effectiveness vary.

Nevertheless, the experiences of older people, their caregivers, and professionals tend to be predominantly positive. The older people and their carers value reablement principles supporting independence [20]. In earlier studies, older people identified the defining and collaborative goal-setting process with the reablement team as a major factor contributing to their sense of self-efficacy and intrinsic motivation [21]. Furthermore, Hjelle [22] indicated that professionals emphasize the importance of engaging in activities that are crucial for older people to perform independently as a contributing factor to their advancement.

In the Netherlands, several reablement programs have been developed in recent years to help older people to remain independent at home. These programs guide care professionals who previously focused on performing ‘tasks for’ older people rather than encouraging their active participation [23]. they align with the reablement model, I-MANAGE, which focuses on improving self-management and participation in meaningful daily activities to support independent living [24]. The I-MANAGE model consists of five phases and identifies six core components: 1) improving assessment and goalsetting, 2) stimulating self-management during meaningful daily activities, 3) optimizing the use of the physical environment, 4) optimizing the use of the social environment, 5) improving interprofessional collaboration, and 6) supporting informal caregivers. In recent years, the Longer Active at Home (in Dutch: Langer Actief Thuis [LAT]) program was developed in southwestern Netherlands (Pruijssers N: Masterthesis Langer Actief Thuis [master], unpublished). It is based on international evidence and the international definition of reablement [12]. LAT is a practical application of I-MANAGE. While initial experiences were positive, the LAT program had not been formally evaluated. This study therefore aimed to assess the feasibility of implementing the LAT program in the Dutch context.

## Method

### Study design

To evaluate the feasibility of the LAT program, we conducted a mixed-methods pilot study with a pre-/post-study design [25]. More specifically, we looked at the five main feasibility aspects according to Bowen [26]: acceptability, demand, implementation, practicality, and limited efficacy. The study was conducted between June 2022 and August 2023 in district nursing teams of five participating organizations. Qualitative and quantitative data on these aspects were collected and analyzed simultaneously. Subsequently, both analyses were compared and the results were integrated to obtain a more complete understanding. These findings were accordingly related to reablement theory.

### Setting

At the time of the study, the LAT program was offered in the south of the Netherlands (West and Middle Brabant) in different care teams of the five organizations. All older people (65 + years) who requested assistance for district nursing care with the participating organizations were potentially eligible for the LAT program, as well as younger clients with multiple chronic conditions. The exclusion criteria for participation in the LAT program were:

- Palliative care in the terminal phase;
- Move to inpatient care within 4 weeks;
- Specific nursing procedures requested, such as bandage or wound care, and full independence in daily activities.

### Intervention: the LAT program

**Fig. 1:**
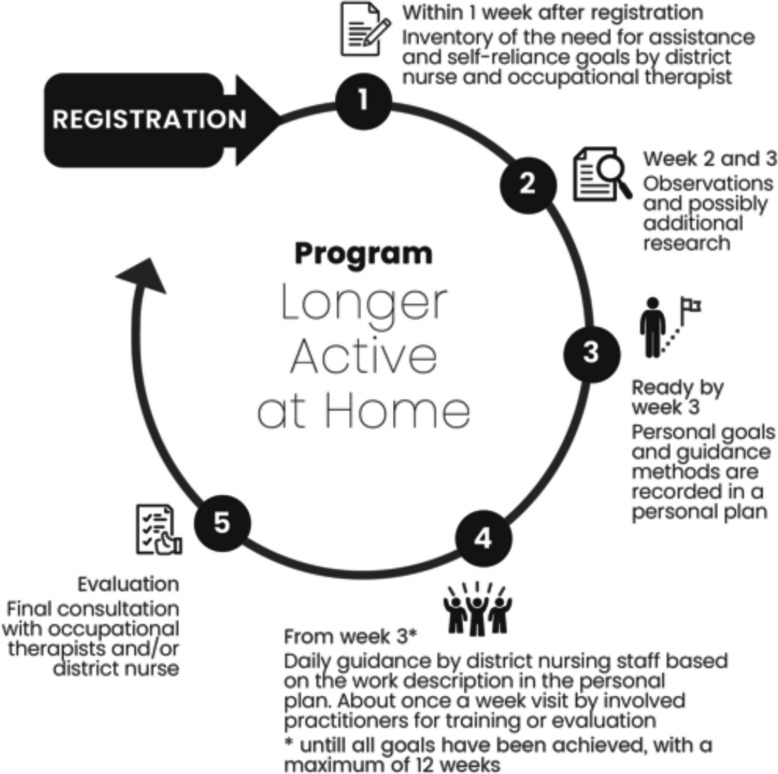
Detailed presentation of the Longer Active at Home (LAT) program. It involves a five-phase process, preceded by the registration of the client, and lasts for a maximum of 12 weeks

The LAT program was delivered by an interdisciplinary team consisting of district nurses (DNs), certified nursing assistants (CNAs), an OT, and a PT. The program consists of five phases and lasts up to 12 weeks (Fig. 1). The full LAT program and the implementation process is described in Appendix 1.

In brief, the LAT program starts with client registration. During the first (telephone) contact between the DN and the client, the LAT program is explained. After registration, an interdisciplinary intake is carried out by the DN and the OT, who broadly ask what is important for the older person to be able to do again. The Canadian Occupational Performance Measure (COPM) is used to identify the older person’s self-reliance goals.

In the second phase, interdisciplinary observations are carried out to identify the older person’s level of functioning. At that point, the strategies to reach the client’s goals are also discussed. Subsequently, specific guidance is discussed with the client and documented as a work instruction in a personal plan. From that moment, each professional of the interdisciplinary team supports the older person in the same way according to the work instruction to reach their personal goals and be as independent as possible in daily activities. Regular interdisciplinary meetings are important to ensure that guidance and work instructions are fine-tuned and adjusted if necessary. After 12 weeks, or when the client has reached their goals, the results are evaluated and long-term care is indicated by the DN where needed.

### Implementation of the LAT program

For the initial 6-month period, organizations were guided in their implementation of the LAT program by a regional implementation coach. This coach facilitated the uniform initiation of the program across all participating organizations. The implementation activities entailed an initial meeting with a core team from the participating organization, comprising representatives from each discipline: OT, PT, DN, and CNA. The formation of a core team within an organization was a prerequisite for starting to implement the LAT program. Together, this core team formulated preconditions for a good start-up in the organization. These preconditions differed between the organizations. The main preconditions focused on supporting interdisciplinary collaboration, such as working in the same electronic care file. After the preconditions were met, there was a kick-off meeting for the entire interdisciplinary team, in which the working method according to the steps of the LAT program was explained to all professionals. During the first 6 months, the core team evaluated the working process every 6 weeks.

### Study population and recruitment

The feasibility evaluation involved two stakeholder groups: the community-dwelling older adults who participated in the LAT program and the professionals who delivered the LAT program.

#### Community-dwelling people who participated in the LAT program

**Fig. 2:**
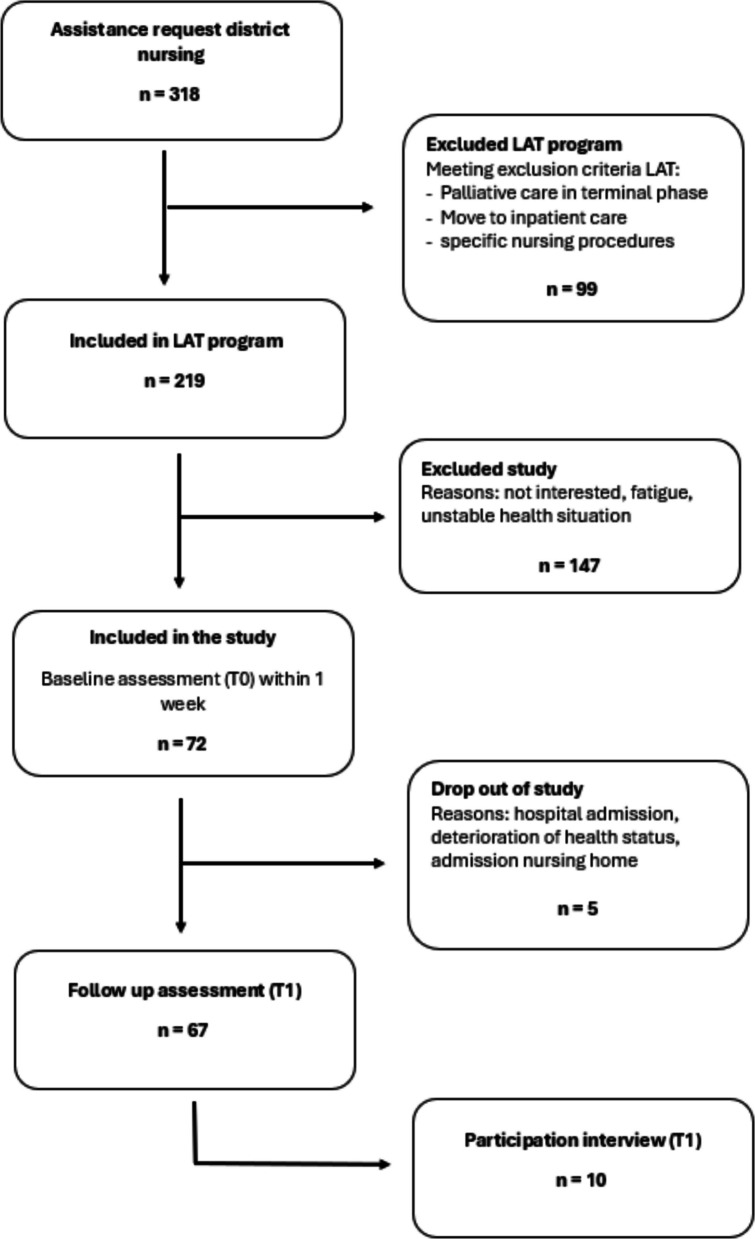
Flowchart of the recruitment process

All people who participated in the LAT program were potential participants for the study. Figure 2 shows the flowchart for the recruitment process. Participants were excluded from the study when they did not formulate a goal based on the COPM or when they were not able to communicate in Dutch. It was decided not to calculate a sample size in advance because this was a pilot study.

All people who were offered the LAT program and met the inclusion criteria of the study were asked by the DN and/or OT to participate in the study at the start of the program. The OT explained the purpose of the study and provided the older person with written information. All older people who were willing to participate enrolled in the study after signing an informed consent form. All participants were enrolled in the quantitative data collection. A subsample was invited to participate in individual interviews. The participants for the interviews were recruited by the OT during the final evaluation of the LAT program between March and August 2023. Besides the general inclusion criteria, the only additional inclusion criterion for participation in the semi-structured interviews was the ability to reflect on and report their experiences with the LAT program. Furthermore, purposive sampling was applied, based on organization, gender, and age in order to enrich the sample and ensure a broad perspective. We included older people across a wide age range (50 to 90 years), with a gender distribution comparable to the quantitative data collection. In addition, participants were recruited from different reablement teams in the different care organizations, mentioned as O1 to O5 (Table 2) to capture a diversity of contexts and experiences. Older people who agreed to participate were called by the researcher, who explained the procedure in greater detail before starting the interview. The participants for the interviews were recruited and included until data saturation was reached, defined as the point at which no new topics or experiences emerged during the interviews.

#### Professionals who delivered the LAT program

The nurses, OTs, and PTs of the teams that delivered the LAT program were asked by the researcher to participate in focus group interviews. The inclusion criterion was that the professional had delivered the LAT program for at least 2 months with at least five clients. With 15 professionals, two focus group interviews could be organized. In allocating the professionals to the two focus groups, the researcher ensured that there was an even distribution of different disciplines across the groups and that professionals from at least two different organizations participated in each group.

### Outcome measures and data collection

This study focused on feasibility testing according to the feasibility framework of Bowen [26], with a focus on the aspects of acceptability, demand, implementation, practicality and limited efficacy. *Acceptability* refers to how professionals and caregivers accept and react to the intervention. *Demand* is the extent to which the program answers to the needs of the target group and if the intervention is likely to be used. *Implementation* addresses intervention delivery to intended participants. *Practicality* refers to the extent to which it is possible to carry out the intervention as intended using the existing means, resources, and circumstances. Finally, *limited efficacy* addresses the potential for success a program demonstrates. Given that this was a pilot study, Bowen’s feasibility aspects of adaptation, integration, and expansion were not considered.

Triangulation was employed, with both qualitative and quantitative data being collected and by consequently relating these findings to reablement theory.

#### Quantitative data

Participants in the LAT program completed a questionnaire providing demographic data and baseline information during their first meeting with the OT and DN. The primary outcome measure was the degree of self-reliance experienced by the older people. This was measured using the Dutch version of the COPM [27]. This instrument measures the self-assessed performance and satisfaction of valued occupations in everyday life within the areas of self-care, productivity, and leisure. The initial COPM evaluation, carried out by the OT, starts with a semi-structured interview during which the older person identifies challenges in daily activities that they consider to be important to be able to do again. The older person was asked to choose up to five relevant activities and to rate separately their performance (COPM-P) and satisfaction (COPM-S) with the performance of each activity on a scale from 1 to 10. A higher score reflects better performance and greater satisfaction. For the re-evaluation at the end of the intervention period, the older person was asked again to rate their performance and satisfaction with their performance in each activity. The difference in the scores indicates the difference in perceived self-efficacy.

The secondary outcome measures to evaluate the initial effects of the LAT program were: the difference in the indicated nursing care time and the difference in average independence score based on a Visual Analog Scale (VAS) from 1 to 10 between the start (T0) and the end (T1) of the LAT program. The instrument to score the independence in daily living was developed specifically for this study (Appendix 2). A higher score reflects the experience of greater independence. A higher independence score and a decrease in indicated district nursing care time was considered indicative of an increase in self-reliance. These data were collected by the OT and the DN at the end of the LAT program. The quantitative data were processed in one of Bowen’s feasibility aspects, namely limited efficacy. A logbook was used to record general data during the data collection period.

#### Qualitative data

Qualitative data regarding Bowen’s five feasibility aspects were gathered during the individual interviews with older people and the focus group interviews with the professionals.

The individual interviews with older people were conducted within 2 weeks after completion of the LAT program by the first author (EB) from March to August 2023. The participants did not know the researcher before the interviews. The focus group interviews were planned to take place between April and June 2023 and lasted approximately 1.5–2 h. The two focus group interviews were conducted by two researchers (EB and TS) with the first author (EB) serving as the moderator. The interviews were guided by a semi-structured interview guide (Appendices 3 and 4) with open and probing questions about the five main feasibility aspects proposed by Bowen [26]. Table 1 describes the data collected method that was used for each feasibility aspect. All interviews were audio-recorded to ensure the completeness and accuracy of the data collected.

**Table 1:** Overview of feasibility measures and interview topics related to Bowen’s [26] areas of focus

Bowen’s areas of focus (description)	Data collection			
	**Source (number)**	**Method**	**Operationalization**	**Timing**
**Acceptability** This relatively common focus looks at how the intended individual recipients—both targeted individuals and those involved in implementing programs—react to the intervention	Sample older people (10)	Individual interviews	VAS score (from 1 to 10) to the question: *How satisfied are you with the LAT program expressed on a scale of 1 to 10?*	Within 2 weeks after completion of the program
	Sample older people (10)	Individual interviews	- Opinion about the program - Experiences with receiving the program and the outcome	
	Sample professionals (15)	Focus group interviews	- Opinion about the program - Experiences with using the program in practice	After a minimum of 2 months of experience with using the program
**Demand** Demand for the intervention can be assessed by gathering data on estimated use or by actually documenting the use of selected intervention activities in a defined intervention population or setting	Sample older people (10)	Individual interviews	- Intention to recommend the program	Within 2 weeks after completion of the program
	Sample professionals (15)	Focus group interviews	- Intention to continue to use the program	After a minimum of 2 months of experience with using the program
	Professionals	Logbook	Actual use - Number of clients who received the program - Reasons for not participating in the program	Throughout the implementation
		Questionnaire	Background characteristics of the participants	
**Implementation** This research focus concerns the extent, likelihood, and manner in which an intervention can be fully implemented as planned and proposed, often in an uncontrolled design	Sample older people (10)	Individual interview	- Description of the experienced implementation of the program - Description of the guidance received by the professionals during the program	Within 2 weeks after completion of the program
	Sample professionals (15)	Focus group interviews	- Description of the experienced implementation of the program - Performance according to the protocol - Success or failure of performance - Changes in content, processes, and activities - Factors affecting implementation ease or difficulty	After a minimum of 2 months of experience with using the program
	Professionals	Logbook	- Number of clients who completed/refused/dropped out - Reasons for refusal and dropout	Throughout the implementation
**Practicality** This focus explores the extent to which an intervention can be delivered when resources, time, commitment, or some combination thereof are constrained in some way	Sample older people (10)	Individual interviews	- Facilitating factors to participate in the program - Barriers to participation	Within 2 weeks after completion of the program
	Sample professionals (15)	Focus group interviews	- Amount/type of resources needed to implement - Preconditions for implementing the program	After a minimum of 2 months of experience with using the program
**Limited efficacy** Many feasibility studies are designed to test an intervention in a limited way. Such tests may be conducted in a convenience sample, with intermediate rather than final outcomes, with shorter follow-up periods, or with limited statistical power	Sample older people (10)	Interview	- Examples of experienced self-reliance	Within 2 weeks after completion of the program
	Sample professionals (15)	Focus group interviews	- Description of the added value of the program for: clients/professionals	After a minimum of 2 months of experience with using the program
	Older people	Questionnaire	- VAS score regarding self-reliance - Described goals in daily activities - Difference in COPM-P/COPM-S scores	Throughout the implementation
	Professionals	Logbook	- Difference indicated district nursing time - Duration of the program	Throughout the implementation

*Abbreviations*: *COPM-P* Canadian Occupational Performance Measure – Performance, *COPM-S* Canadian Occupational Performance Measure – Satisfaction, *LAT* Longer Active at Home, *VAS* Visual Analog Scale

### Data analysis

#### Quantitative data

The quantitative data were analyzed using SPSS Statistics for Windows Version 29 (IBM Corp., Armonk, NY, USA). Missing data were identified through initial data screening and descriptive statistics. Analyses were conducted using all available data for each person. Descriptive statistics were performed to present baseline characteristics. The mean and standard deviation (SD), or median and interquartile range in case of a non-normal distribution, were determined for the continuous characteristics, and the frequency and percentage were determined for the categorical characteristics. Selective dropout was checked by comparing the characteristics of the dropouts with the non-dropouts by using the Mann–Whitney U test for continuous variables and the chi-square test for categorial variables.

A multilevel linear regression analysis was used to assess the difference between the first and second measurements for the outcomes (COPM-P, COPM-S, VAS, and district nursing time). A random intercept model with time as a factor and potential confounders (age, gender, the number of conditions, and indicated district nursing care) was used. A *p*-value < 0.05 was considered to be statistically significant for all analysis, based on two-sided testing.

#### Qualitative data

The interviews were recorded, transcribed verbatim, and anonymized. Analysis was performed using the qualitative data analysis software ATLAS.ti Windows (Version 24.0.0.). A reflective thematic analysis (RTA) approach was used, following the steps identified by Braun and Clarke [28]. The client interviews and focus group interviews were analyzed separately.

All transcripts were carefully listened to and read by the first author (EB) for familiarization. Two researchers (EB and IM) independently coded three interview transcripts. They created open codes that were close to the original transcript text. The codes were compared for similarities and differences were discussed. They agreed on further procedures for coding, developing potential initial themes. The remaining interviews were coded by EB in a similar manner. Relationships between codes were identified, and these codes were grouped together. Similar codes were organized under potential initial themes. These codes and potential initial themes were jointly discussed and refined by the researchers (EB and IM). This resulted in confirmation of the initial themes separately for the two different stakeholder groups; these themes were discussed in project team meetings with all authors. During these meetings, the analysis was refined and a consensus was reached regarding four overarching initial themes for each stakeholder group.

The analysis was an iterative process, during which initial codes were recoded and relationships and initial themes were revised. In the next step, the initial themes for both groups were compared and discussed with the entire team. After extensive analysis, the researchers (EB and IM) identified three overarching themes, which were then discussed with and confirmed by the wider research team. Thereafter, the feasibility was deductively analyzed and described in accordance with Bowen’s categories, as outlined in Table 1. After analyzing the quantitative and qualitative data simultaneously, we triangulated our findings by relating them to each other and to the theoretical concepts of reablement.

## Results

### Study participants

Table 2 provides the baseline characteristics for the 72 older people who participated in the study. Five older people stopped with the LAT program and dropped out of the study. The reasons for dropout were hospital admission (*n* = 1), deterioration of the health status (*n* = 3), and admission to a nursing home (*n* = 1). There were no significant differences in the characteristics of dropouts and completers. Ten older people participated in the interviews after completing the LAT program. Their sociodemographic characteristics are also presented in Table 2.

**Table 2:** Characteristics of the participants

Variables	Quantitative data	Qualitative data
Gender, n (%)	72 (100)	10 (100)
*Female*	51 (70.8)	8 (80)
Average age (years), mean [SD]	79.2 [8.8]^a^	77.6 [12.2]
Organization, n(%)	72 (100)	10 (100)
O1	37 (51.4)	6 (60)
O2	19 (26.4)	2 (20)
O3	8 (11.1)	1 (10)
O4	6 (8.3)	
O5	2 (2.8)	1 (10)
Housing, n (%)	59 (100)	
*Apartment*	40 (67.8)	8 (80)
*House*	19 (32.2)	2 (20)
Civil status, n (%)	59 (100)	
*Living together*	22 (37.3)	7 (70)
*Living alone*	37 (62.7)	3 (30)
Received allied health intervention at the start of the LAT program, n (%)	68 (100)	
*Occupational therapy (%)*	6 (8.8)	
*Physiotherapy (%)*	30 (44.1)	
*Other (%)*	4 (5.6)	
Received domestic support at the start of the LAT program, n (%)		
Yes	47 (67.1)	
Indicated district nursing time in minutes per week, mean (SD)	169 (105.32)	
Independence score at T0, mean (SD)	5.6 (1.66)	
COPM-P score, mean (SD)	4.49 (1.73)^b^	
COPM-S score, mean (SD)	4.53 (1.66)^c^	

The superscript numbers indicate the number of people with missing values: ^a^ = 1 person, ^b^ = 2 people, and ^c^ = 3–5 people

*Abbreviations*: *COPM-P* Canadian Occupational Performance Measure – Performance. *COPM-S* Canadian Occupational Performance Measure – Satisfaction, *LAT* Longer Active at Home, *SD* standard deviation, *T0* start of the intervention

Fifteen professionals participated in the two focus group interviews: DNs (*n* = 4), a nurse (*n* = 1), a student nurse (*n* = 1), CNAs (*n* = 3), OTs (*n* = 4), and PTs (*n* = 2). All professionals had between 2 months and 1 year of experience with implementing the LAT program.

### Quantitative results

#### Goals

**Fig. 3:**
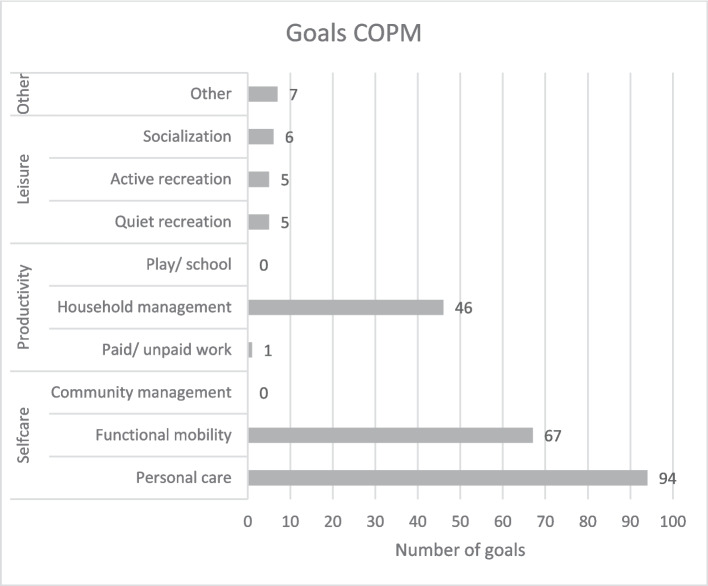
The bar chart presents the classification of the goals set by the participating older people. The bar chart displays the number of goals set within each category of the Canadian Occupational Performance Measure (COPM; i.e., leisure, productivity, and self-care). Each category is also divided into sub-categories

All 72 older people formulated 1 to 5 goals (231 goals in total). The goals were mainly related to personal care (*n* = 94, 40.7%), functional mobility (*n* = 67, 29.0%), household management (*n* = 45, 19.5%), and leisure (*n* = 16, 6.9%). The details of distribution of the COPM goals are shown in Fig. 3.

For all the outcomes, there was a clinically significant increase in the score between T0 and T1. Regarding their goals, after the LAT program older people experienced better performance (COPM-P: ∆ = 3.10, 95% confidence interval 2.68–3.52) and greater satisfaction about their performance (COPM-S: ∆ = 3.18, 95% confidence interval 2.71–3.64). In addition, they experienced more independence (∆ = 2.11, 95% confidence interval 1.72–2.50) and needed on average 2 h less district nursing care (Table 3).

**Table 3:** Mean difference of outcomes between baseline (T0) and the follow-up assessment (T1)

Variables	T0 (*N* = 72)		T1 (*N* = 67)		Mean difference (T1 – T0)	95% confidence interval of the mean difference		*P*-value
	**N**	**Mean (SD)**	**N**	**Mean (SD)**		**Lower limit**	**Upper**	
COPM-P	70	4.49 (1.73)	66	7.57 (1.29)	3.10	2.68	3.52	< 0.01
COPM-S	67	4.53 (1.66)	63	7.68 (1.38)	3.18	2.71	3.64	< 0.01
Independence score	72	5.60 (1.66)	66	7.70 (1.14)	2.11	1.72	2.50	< 0.01
Indicated district nursing time (minutes/week)	72	169.13 (105.32)	65	39.78 (86.51)	−126.50	−149.17	−103.84	< 0.01

This table presents the mean scores for each outcome measure and the mean difference between the start and follow-up measurements with the 95% confidence interval and significance presented. For T0 and T1, the “N” column indicates the number of older people for whom data were available to calculate the mean and SD

*Abbreviations*: *COPM-P* Canadian Occupational Performance Measure – Performance, *COPM-S* Canadian Occupational Performance Measure – Satisfaction, *LAT* Longer Active at Home, *SD* standard deviation, *T0* start of the intervention

### Qualitative results

The qualitative analysis revealed three central themes that illustrate the results and challenges of implementing the LAT program.

#### Theme 1: necessary and desirable shift in mindset toward self-reliance

This theme highlights the shift in mindset required to move from providing care to enabling independence, a transition seen as both necessary and desirable.

The professionals emphasized that LAT aligns well with broader social trends in the Netherlands, where there is a growing emphasis on enabling older adults to remain in their homes for as long and independently as possible. They noticed that the current clients want to continue to live in their own home for as long as possible. The new LAT approach encourages a transformation from a focus on “doing for” to “doing with,” redefining the role of care workers from providers of care to enablers of independence.

*For the client, I think the language you speak is particularly important. Like “we’re not coming to huh, doing it for you, but doing it with you.” Yes, we have to name it differently…. Yes, socially, we have to try to make a move there too. That it is not like “Oh just do it with the district nursing team and they’ll solve it…”* (Occupational Therapist; FG2)

Initially, this shift in mindset posed challenges for the different teams, as CNAs encountered resistance, both within themselves and among colleagues, when adopting the new approach. The professionals mentioned that it took time for the team to make the shift in mindset toward supporting self-reliance. The resistance encountered was primarily due to the fact that the CNAs were used to providing care.

*In the beginning, you really have the idea of, “Oh, but I’m standing here doing nothing.” And that switch has now been flipped. Now I think, “No, I’m not doing nothing, I’m watching what the other person can do.”* (Certified Nursing Assistant; FG2).

Success stories of “*providing care with hands behind their back*” played a crucial role in overcoming resistance, leading toward the shift in mindset among care workers.

*They find it very difficult to actually not be able to offer anything, but then you see, they still have a success experience that you can still get very nice results with the hands behind your back…* (Occupational Therapist; FG2)

The interdisciplinary nature of the LAT program facilitated this transformation. By integrating insights from different disciplines, teams worked together on self-reliance with a shared ownership that even resulted in increased job satisfaction among the care workers involved. The role of management and organizational support was also identified as a contributing factor in this regard.

*We really make each other better. We can set goals that we can work on together, so it does encourage us to work together.* (District Nurse; FG1)

For the older people, the LAT program was a transformative experience. The participating older people reported a distinctive approach employed by CNAs. They noticed that the nursing assistants no longer took over tasks, but rather stood beside them to guide them towards self-reliance. They also highlighted the provision of support throughout this process.

“*It was good to have someone there to support me. You have support even if they just stand there.”* (Client 09)

However, the process was not without its challenges. The professionals and older people noted that family members, some clients, and informal caregivers sometimes resisted the LAT approach, either expecting more professional care or underestimating the capabilities of the client. This underscored the importance of involving the entire social network in promoting self-reliance while keeping the client’s needs central to the approach.

*With clients who already receive care, we do notice resistance… it’s just really hard! And what I also hear very often from these people is “yes, I’ve worked hard all my life, I’m entitled to this.*” (Certified Nursing Assistant; FG 1)

Despite these challenges, the LAT program was seen as a valuable tool for initiating societal conversations about fostering independence and self-direction among older adults in the Netherlands. By supporting self-management skills and promoting confidence, the LAT program paves the way for sustainable, client-centered care.

*…but actually we start with LAT with everyone and explain what LAT means and how we work, because that is simply our way of working to keep that control with the client for as long as possible.* (District Nurse; FG1)

#### Theme 2: collaboration – key to success, but a challenge in practice

Effective collaboration across diverse disciplines is important for achieving success. The second theme examines the benefits of interdisciplinary collaboration while acknowledging the practical challenges it entails.

According to the professionals, the interdisciplinary collaboration that characterizes the LAT program is a key factor in its success. The vast majority of the professionals involved in the program indicated that interdisciplinary collaboration plays a crucial role in achieving positive outcomes. An added value was to carry out interdisciplinary intakes and observations, because different professional perspectives lead to a broader and more cohesive approach. It was mentioned that all disciplines complement each other in this collaboration.

*I think the key to working well together is to put our heads together and see who’s got what expertise. I really do think that’s an added benefit of LAT.* (Occupational Therapist; FG2)

In this respect, the findings show that each core discipline has its own role. For example, DNs were described as the “cornerstones” of LAT teams, acting as the primary link between clients, general practitioners (GPs), and other team members. OTs were seen as catalysts for self-reliance, offering innovative strategies and crafting work instructions that guided the care process. The involvement of the OT from the start of the care request was therefore considered important. Meanwhile, PTs focused on creating conditions to enable independence during functional training in the client’s home environment, while CNAs executed the tailored plans. Moreover, OTs regarded them as sparring partners when delineating the specifics of work instructions. Altogether, these inter disciplinary teams created greater ‘synergy’ in organically influencing their counterparts.

Team access to the client’s electronic care file was considered to be an important component of the working process. All professionals expressed that their teams worked with the work instructions in the client’s electronic care file. This was generally well maintained and guided the support of the client for all disciplines involved. These work instructions were considered to be a key component in the success of the intervention.

*If then that work instruction is so well described, you are going to do it exactly the same way.* (Certified Nursing Assistant; FG2).

Additionally, the interdisciplinary meetings were considered to be an integral component of the intervention, facilitating coordination between disciplines in the delivery of client support.

Older people recognized the benefits of this collaborative approach. Some of them mentioned the collaboration between the disciplines.

*… not necessarily just from the district nurse or just from the occupational therapist, but I think the program together—or the collaboration together—is conducive to the patient's recovery.* (Client 01)

However, not all older people were fully aware of the interdisciplinary collaboration, indicating a requirement to improve communication about team roles.

Despite its benefits, collaboration was not without obstacles. Busy schedules and the need for care to be started with short notice often prevented interdisciplinary intakes by DNs and OTs, leading to fragmented assessments. Inconsistent documentation sometimes hindered the progress of the process. Moreover, different electronic care systems within and between organizations made sharing information difficult. The professionals emphasized the importance of organizational support in overcoming these logistical barriers.

*[Support is important] Well, especially that the organization carries it along [and tell us] … This is how we are going to work…* (Occupational Therapist; FG2).

#### Theme 3: guidance toward self-reliance – boosting confidence and reducing care dependency

The third theme focuses on the tangible outcomes of the LAT program, highlighting how it fosters independence and self-confidence among older people and reduces their reliance on professional care. The older people mentioned that the LAT program empowered them to regain control over their lives and to (re)gain self-management skills. The professionals observed significant increases in the clients’ self-confidence and ownership as they achieved their personal goals. This newfound independence often led to a reduction in the demand for district nursing services, allowing the district nursing teams to provide care to other clients.

*I think if you get people back to doing things for themselves, it also ensures that they participate in society again, are healthier at the end of the day and therefore use less district nursing.* (Physiotherapist; FG1)

The older people endorsed these sentiments and described their path to independence. Through the LAT program, they acquired the ability to perform daily tasks independently or with the assistance of aids. Their narratives align with the quantitative results: They illustrate the profound impact of the LAT program in promoting self-reliance and self-confidence in doing activities that are important to them. The older adults also expressed an improvement in quality of life as they experienced the freedom of being independent again.

*Now that I perform these tasks independently, it requires more time, but I take my time. I have the time for it and I am content with this.* (Client 09)

#### Feasibility according to Bowen

Our findings can be categorized under Bowen’s five main feasibility categories: acceptability, demand, implementation, practicality and limited efficacy. Table 4 gives an overview of the results of the analysis presented from these categories.

**Table 4:** Overview of the qualitative results related to Bowen’s [26] areas of focus

Area of focus	Sample outcomes of interest	Professionals	Older people
**Acceptability**	- Satisfaction - Intent to continue to use - Perceived appropriateness - Fit within organization culture - Perceived positive or negative effects on organization	- Conducting LAT contributes to a shift in mindset from doing for to self-reliance - It takes time for professionals to make the shift in mindset - Successful experiences in “caring with the hands behind the back” encourage the shift in mindset - Interdisciplinary collaboration benefits professionals; they learn from each other - It is important to involve family and the informal network to avoid resistance	- Satisfied with the way LAT works - Appreciate the approach at home - Gaining self-confidence through the professionals’ guidance - Like being able to be (more) independent again - Consider LAT to be valuable and would participate again
**Demand**	- Actual use - Expressed interest or intention to use - Perceived demand	- LAT fits within social developments in the Netherlands - Some older people and their families have different expectations of professional care, especially when they already receive professional care - In doing so, LAT is experienced as a means to conduct the social conversation about self-reliance with older people	- Most older people appreciate being guided so that they are able to do the activities that are important to them
**Implementation**	- Degree of execution - Success or failure of execution - Amount and type of resources needed to implement - Factors affecting the ease or difficulty of implementation -Efficiency, speed, or quality of implementation	- The strength of the intervention lies in interdisciplinary collaboration, with each discipline having its own focus - The core team consists of an occupational therapist, district nurse, and certified nursing assistant. In most organizations, the physiotherapist is also part of the core team - The main preconditions are focused on supporting interdisciplinary collaboration - An implementation coach ensures uniform implementation - Busy schedules and limited time of the professionals hinder interdisciplinary collaboration	- Experience guidance and support by the various professionals to function as independently as possible - Some clients experience collaboration between the disciplines
**Practicality**	-Positive/negative effects on target participants -Ability of the participants to carry out intervention activities -Cost analysis	- Emphasize the added value of interdisciplinary collaboration during intake and observations, although this did not succeed in about half of the cases due to busy schedules -COPM grading is difficult for some clients, but the COPM goals give a clear direction and the scores provide a lot of information to the client, thus improving their involvement - Working with the same client file and good reporting are important for the program to succeed	
**Limited efficacy**	- Intended effects of program or process on key intermediate variables - Effect size estimation - Maintenance of changes from initial change	- Stronger ownership among clients as they work on their own goals - LAT contributes to older people’s self-management - Professional care can be phased out for some older people - Self-reliance and self-confidence increase among clients	- Become (more) independent by learning to perform activities in a different way or with help of aids - Most of the older people are able to return to doing the activities that are important to them independently or with less professional care

*Abbreviations*: *COPM* Canadian Occupational Performance Measure, *LAT* Longer Active at Home

## Discussion

This study evaluated the feasibility of the LAT reablement program in the Dutch healthcare context. The findings demonstrate that the LAT program is feasible according to Bowen’s [26] aspects of acceptability, demand, implementation, practicality, and limited efficacy. We found that the LAT program is received positively by both older people and professionals in the context of Dutch social developments. Older people as well as professionals displayed and experienced a statistically significant increase in self-reliance and self-confidence after completing the LAT program. Additionally, older people required on average less than 2 h of district nursing care per week. Furthermore, professionals noted a shift in mindset from a “doing for” to a “doing with approach” facilitated by the program and interdisciplinary collaboration.

We found statistically significant and clinically relevant changes. The COPM-P and COPM-S scores increased by more than 3 points, exceeding the cut-off for a clinical relevant change, suggested by Law et al. [29] and consistent with the stricter cut-off for clinical relevant change suggested by Tuntland et al. [30]. The qualitative findings reinforced the clinical relevance, as participating older people as well as professionals indicated that they experienced and observed an increase in self-reliance and self-confidence in older people who completed the LAT program. Accordingly, we triangulated our findings with the theoretical concepts and findings from the literature, which confirmed each other. Our qualitative and quantitative results on limited effectiveness as well as other qualitative studies in the literature indicate greater independence in daily activities, autonomy, and self-management of older people in their own homes and social environment as a result of reablement interventions [21, 31, 32].

Consistent with the enhancement in self-reliance and self-confidence, there was a statistically significant decline in district nursing time in the present study, which can be seen as a preliminary indication that the LAT program can lead to cost savings. However, further research is required to expand upon these findings. Nevertheless, our findings align with earlier studies showing that reablement reduces home care use and duration of professional visits, with effects lasting from 6 months to several years [33–36].

The professionals we interviewed experienced a shift in their mindset from a “doing for” to a “doing with” approach that was facilitated by the LAT program. Other researchers have reported that, to implement reablement, a shift in mindset is necessary for all those involved. This has been mentioned as a process of cultural change and identified as a time-consuming process [23, 37]. Leadership, re-learning, re-education, and success are described as common elements in successful culture change [38]. Mouchaers et al. [32] also mentioned these elements in relation to the implementation of another Dutch reablement intervention. In our study, the professionals also highlighted that a clear vision, effective leadership, well-defined steps, and positive success experiences can contribute to a shift in mindset. The Dutch report ‘A good day on your own’ (in Dutch: Een goede dag op eigen Kracht) [39] also describes the importance of behavioral change among healthcare professionals in implementing reablement. Furthermore, this report distinguishes between the philosophy of reablement and reablement intervention programs. Reablement intervention programs provide a structured framework that can act as a catalyst to accelerate the philosophy of reablement. Our findings support this view: The professionals found that the structured LAT program provided them with the tools necessary for effective implementation of a “doing with” approach. This, together with the success experienced with the new way of working, helped them to make the shift in mindset.

Interdisciplinary collaboration was also identified as essential. Consistent with other studies, professionals valued working toward shared goals as motivating [32, 40]. Also the role of management was considered by other studies to be important in facilitating the implementation of reablement [32, 40]. Dutch policy advocates increased collaboration between various stakeholders across different domains to ensure the delivery of suitable, personalized care [10, 41]. Interdisciplinary or interprofessional collaboration is encouraged. In practice, interdisciplinary collaboration can present certain challenges [42]. Professionals act within the boundaries of their own profession, and tasks are coordinated and distributed among the professionals involved after mutual consultation [43]. Processes such as boundary-blurring and task-shifting, where responsibilities are shared across roles, may enhance teamwork and holistic care [43–45]. Interprofessional collaboration requires a shared way of thinking as well as an interprofessional identity, within which the conversation is about the unique role of each discipline within interprofessional collaboration [44].

While interdisciplinary or interprofessional collaboration is recognized as a key component of reablement, there is a lack of understanding regarding the specific roles of various disciplines and the elements that contribute to successful interdisciplinary collaboration. For example, what stimulates professionals to develop an interprofessional identity and to take part in a boundary-blurring process? How can roles be specified in the interdisciplinary team and what does the boundary-blurring process mean for the collaboration? In short, what works, for whom, under what circumstances, and how?

### Strengths and limitations

A major strength of this study is its use of a mixed-methods design, which facilitated the creation of a comprehensive overview and the triangulation of findings. Moreover, Bowen’s [26] framework and the experiences of professionals and older people complemented each other, enhancing the reliability of our study. Another strength is the use of the COPM as the primary outcome measure, which enabled the measurement of a person-centered outcome measure, self-reliance [27]. The sample size for this pilot study is also a strength, including 72 participants with a low drop-out rate of 7%. Additionally, the diversity of participants across different organizations, including both older adults and professionals, further strengthened the study and supported triangulation of findings.

In addition, our paper provides a detailed description of the structure and implementation of our reablement program. Although there is a growing international interest in reablement, in many countries the approach is still emerging. This paper may equip professionals with concrete tools to apply reablement strategies in real-world settings, bridging the gap between research and practical application.

Several limitations should be acknowledged. First the study included a limited set of outcome measures, which does not cover the full range recommended by Tuntland et al. [45], including performance in activities of daily living, physical functioning, health-related quality of life, and social connectedness [13]. Second, although we determined differences in care use based on care time, we did not measure the number of visits per participant or actual time spent on treatment. Inclusion of such data could have provided additional insights. Third, selection bias may have occurred, as participating older people were recruited by DNs and OTs, which may have led to the inclusion of more motivated participants.

Overall, despite these limitations, the study’s methodological rigor, diverse sample, and mixed methods approach provide robust and valuable insights into the feasibility and outcomes of the LAT program in the Dutch context.

### Practical implications

Future research could focus on investigating the long-term effects and the cost-effectiveness of the LAT program. It would add value to measure actual hours of care and treatment and to include a quality of life outcome measure in addition to outcome measures focused on self-reliance, such as the COPM. To enhance the comparability of research findings, we recommend that future reablement programs are based on the same conceptual model as the LAT program, which is based on the I-MANAGE model, and that reablement studies make use of comparable outcome measures. Another focus for future research could be to unpack the black box of interdisciplinary collaboration in reablement programs. Systematic examination of the literature and consultation with relevant professionals is required to unravel the mechanisms of interdisciplinary and interprofessional collaboration, and to identify what circumstances influence these collaborations, and the roles involved.

## Conclusion

This is the first Dutch feasibility study on reablement which combined differences is district nursing time with quantitative and qualitative findings on self-reliance. The study demonstrates that the LAT program is a feasible interdisciplinary program that contributes to the self-reliance and self-confidence of older people, while reducing district nursing time. The program also supports a cultural shift among professionals from a focus on “doing for” to “doing with” older people, redefining their role of from providers of care to enablers of independence. The LAT program aligns with broader social trends in the Netherlands, emphasizing support for older people to live in their homes as long and independently as possible.

## Supplementary Information

Supplementary Material 1.

## Figures and Tables

**Fig. 4:**
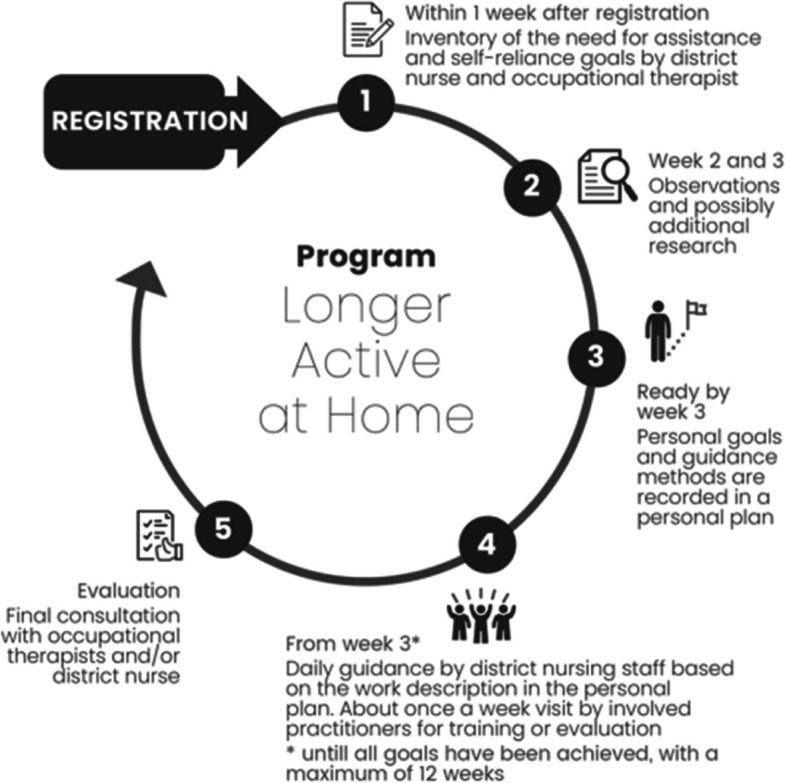
Infographic of the Longer Active at Home (LAT) program describing the steps taken during the execution of the program

## Data Availability

Thedatasets used and/or analyzed during the current study are available from the corresponding author upon reasonable request.

## Appendix 1

### The Longer Active at Home (LAT) program: description of the content and implementation

The LAT program is based on the international reablement definition [12] and aims to help older people to increase or maintain their independence in meaningful activities of daily living at their place of residence and to reduce their need for long-term care.

The LAT program has a maximum duration of 12 weeks and consists of five consecutive phases: 1) initiation/intake, 2) observation, 3) care plan, 4) care delivery, and 5) evaluation (Fig. 4).

#### Inclusion/exclusion

All people (65 + years) who request assistance for district nursing care are eligible for the LAT program. Younger clients with multiple chronic conditions are also eligible for the LAT program. The exclusion criteria for participation in the LAT program are:

- Palliative care in the terminal phase;
- Move to inpatient care within 4 weeks;
- Specific nursing procedures requested, such as bandage or wound care, and full independence in daily activities.

#### Phase 1: initiation

During the first (telephone) contact between the district nurse (DN) and the older person, the LAT program is explained to the client. A first interview will be scheduled within a week and will be conducted by the DN and the occupational therapist (OT). The OT uses the Canadian Occupational Performance Measure (COPM) to conduct the interview and to set a maximum of five meaningful goals with the client. The DN supplements the interview with questions from a broader community perspective. It is jointly assessed whether other disciplines, such as a physiotherapist (PT), should be involved in the LAT program. After the intake, the DN indicates the district nursing care time required and creates a personal plan for the client in his or her electronic client file. The DN fulfills the coordinating role in the LAT program and works closely with the OT.

#### Phase 2: observation

During a joint observation phase, there are (interdisciplinary) observations by professionals that are involved in the client’s goals. During the observations, the client’s needs are assessed to determine the type of guidance required to work on their goals. The purpose of the joint observations is to create a common starting point. In addition, the joint observations contribute to interdisciplinary learning, which plays an important role in on-the-job-training.

#### Phase 3: personal plan

The DN is responsible for creating a personal plan in the client’s electronic file. The client’s personal goals are included in this plan. The possible interventions and actions to achieve the client’s goals are discussed by the LAT team. The starting point is to work toward independent functioning, possibly supported with (technological) aids. Based on the observations, the OT and/or PT adds a work description to the client’s personal goals in their electronic file. The work description shows in detail: (1) what the client can do by themself; (2) what (verbal) guidance is needed; and (3) which help/support is needed. The involvement and possibilities of informal caregivers and the client’s social network is also described in the personal plan. The intensity of the program depends on the care needs of the client and their preset goals, which may require a higher intensity of in the beginning but less at the end when (sub-) goals are (partly) reached.

#### Phase 4: care delivery

The interdisciplinary team delivers care and support as described in the personal plan. The OT and PT are responsible for adjusting the work description when guidance changes. The DN is responsible for adjusting the newest work description in the care plan. The OT and PT are also responsible for assisting with (re)learning and practicing meaningful daily activities and stimulating participation in daily life. The OT also assesses which assistive devices or home modifications may need to be implemented to optimize self-reliance and participation. The certified nursing assistants are responsible for working in accordance with the current work description in the care plan. The DN is responsible for supporting and managing the client’s personal care needs. While supporting meaningful daily activities, the clients self-management should be stimulated by practicing activities and ensuring that tasks are not taken over by care professionals or informal caregivers.

#### Phase 5: evaluation

The performance of daily activities and the care process is structurally evaluated in interdisciplinary LAT team meetings, which take place every 2 weeks and involve a discussion of what (further) steps are needed to support the client in achieving the formulated goals. Based on the conclusions drawn during these meetings, the personal plan and/or work description is continued, adjusted, or terminated. Changes in guidance always take place in consultation with the client. The LAT program ends when the client has achieved his or her goals or after a maximum of 12 weeks. At the end of the program, a formal evaluation of the client’s goals takes place using the COPM, including scoring the performance and satisfaction within the activities. When formal care is still needed after completion of the LAT program, the DN assesses the level of care and provides an indication for this care.

## Abbreviations

CNA: Certified nursing assistant
COPM: Canadian Occupational Performance Measure
COPM-P: Canadian Occupational Performance Measure– Performance
COPM-S: Canadian Occupational Performance Measure– Satisfaction
DN: District nurse
FG: Focus group
GP: General practitioner
LAT: Langer Actief Thuis (Longer Active at Home)
OT: Occupational therapist
PT: Physiotherapist
VAS: Visual Analog Scale
WOZO: Wonen, Ondersteuning en Zorg voor Ouderen (Living, Support and Care for the Elderly)
