# Supplementary material for: Positive changes after a reablement program for older people in the Netherlands: a mixed-methods feasibility study
Source: BMC Geriatr. 2026 Apr 23;26:855. doi: 10.1186/s12877-026-07460-4 (PMC13281620; doi:10.1186/s12877-026-07460-4)
Supplement: Supplementary file 1 — Supplementary Material 1. [file 12877_2026_7460_MOESM1_ESM.docx]

**Appendix 2: Visual Analogue Scale independence**

How do you rate your independence in daily living on a scale from 1 to 10 where a higher score reflects the experience of a greater independence?

1 - 2 - 3 - 4 - 5 - 6 - 7 - 8 - 9 - 10

Totally Totally

dependent independent

on help of others

of others

**Appendix 3: Interview guide for the interviews with older people**

1. **Bowen: Acceptability**
2. Can you say something about your experiences with the LAT program?
3. How satisfied are you with the program (rating from 1 to 10)?
4. What did you appreciate most during the program?
5. What did you dislike?
6. What goals did you work on during the program?
7. What did you do to reach the goals?
8. **Bowen: Limited effectiveness**
9. You have learned/practiced several things recently. Can you give an example of these?
10. Since your participation, are there any changes in the activities you do independently again?
11. Which changes?
12. How do you feel about this?
13. **Bowen: Demand**
14. Would you participate again if the program was offered to you? Why?
15. For which people in your environment would this program also be suitable?
16. **Bowen: Implementation**
17. Can you describe which professionals guided you during the LAT program and what they practiced or discussed with you during the program?
18. What did the professionals do to help you to reach your personal goals?
19. What did you notice about the collaboration between the district nurse, the nursing assistants, the physiotherapist, and the occupational therapist?
20. **Bowen: Practicality**
21. What helped you to work on your personal goals during the LAT program?
22. What sometimes operated as a hindrance to this?

**Appendix 4: Interview guide for the professional focus groups**

1. **General introduction**
2. Can you briefly introduce yourself, indicating your job title, where you work, and how long you have been involved with LAT?
3. **Bowen: Acceptability**
4. How would you describe your experience of the new way of working according to LAT?
5. What do you experience as a difference between how you worked before and how you work with LAT?
6. Which part of LAT do you find most valuable?
7. Which part of LAT would you like to design differently?
8. **Bowen: Limited effectiveness**
9. What is the added or missing value of LAT, from the perspective of:
10. The client?
11. The professional?
12. The organization?
13. Society?
14. Are you satisfied with the effects you have seen in practice?
15. Can you mention some positive effects?
16. Can you mention some negative effects?
17. Which client groups do you think benefit the most of the program? Which client groups do not benefit of it?
18. **Bowen: Demand**
19. To what extent do you feel the LAT program aligns with current social developments?
20. Do you intend to continue using LAT in the future?
21. What measures should be taken to ensure the program’s ongoing success?
22. **Bowen: Implementation**
23. Can you describe how you work according to LAT? Which steps are followed?
24. Do these steps match the description given in the kick off meeting?
25. Which changes are made and why?
26. What are the key elements required for effective implementation of LAT?
27. **Bowen: Practicality**
28. What factors contributed to the success of implementing LAT?
29. What factors do you experience as hindering in the implementation of LAT?
30. What preconditions are necessary to carry out LAT?
31. Who or what can help you to organize these preconditions?
